# Early Versus Late Recurrence in Olfactory Neuroblastoma: A Multi‐Institutional Analysis of Predictive Risk Factors

**DOI:** 10.1002/alr.70216

**Published:** 2026-07-09

**Authors:** Alexandria Harris, Anthony Tang, Nicholas Fung, Joao Paulo Almeida, Pierre‐Olivier Champagne, Juan Fernandez‐Miranda, Paul A. Gardner, Peter Hwang, Aristotelis Kalyvas, Chirag Patel, Zara M. Patel, Maria Peris Celda, Carlos D. Pinheiro‐Neto, Olabisi Sanusi, Carl H. Snyderman, Brian D. Thorp, Jamie J. Van Gompel, Georgios Zenonos, Nathan T. Zwagerman, Diana Bell, Edward C. Kuan, Eric W. Wang, Mathew Geltzeiler, Garret Choby

**Affiliations:** ^1^ Department of Otolaryngology—Head and Neck Surgery University of Pittsburgh Medical Center Pittsburgh Pennsylvania USA; ^2^ Department of Neurological Surgery Indiana University School of Medicine Indianapolis Indiana USA; ^3^ Department of Neurological Surgery Universite Laval Quebec City Quebec Canada; ^4^ Department of Neurological Surgery Stanford University Palo Alto California USA; ^5^ Department of Neurological Surgery NYU Langone Health New York New York USA; ^6^ Department of Otolaryngology—Head and Neck Surgery Stanford University Palo Alto California USA; ^7^ Department of Neurological Surgery University of Toronto Toronto Ontario Canada; ^8^ Department of Otolaryngology—Head and Neck Surgery Loyola University Medical Center Maywood Illinois USA; ^9^ Department of Neurological Surgery Mayo Clinic Rochester Minnesota USA; ^10^ Department of Otolaryngology—Head and Neck Surgery NYU Langone Health New York New York USA; ^11^ Department of Neurological Surgery Oregon Health & Science University Portland Oregon USA; ^12^ Department of Otolaryngology—Head and Neck Surgery University of North Carolina Chapel Hill North Carolina USA; ^13^ Department of Neurological Surgery University of Pittsburgh Medical Center Pittsburgh Pennsylvania USA; ^14^ Department of Neurological Surgery Medical College of Wisconsin Milwaukee Wisconsin USA; ^15^ Department of Pathology and Laboratory Medicine University of Pittsburgh Pittsburgh Pennsylvania USA; ^16^ Department of Otolaryngology—Head and Neck Surgery University of California Irvine California USA; ^17^ Department of Otolaryngology—Head and Neck Surgery Oregon Health & Science University Portland Oregon USA

**Keywords:** esthesioneuroblastoma, metastasis, olfactory neuroblastoma, recurrence, sinonasal malignancy, skull base, staging, surveillance

## Abstract

**Background:**

Olfactory neuroblastoma (ONB) exhibits variable recurrence patterns, with significant late recurrences occurring years after treatment. We investigated cutoff periods to define late recurrence and identified predictors that distinguish patients at risk of late recurrence from those considered cured.

**Methods:**

From a multi‐institutional cohort of 219 ONB patients treated at nine centers, we analyzed 77 patients with documented recurrence. Using a 48‐month cutoff to define early (< 48 months) versus late (≥ 48 months) recurrence, we evaluated 35 clinical, anatomic, and treatment variables using Firth penalized logistic regression across three comparisons: (1) late versus early recurrence timing, (2) late recurrence versus no recurrence, and (3) early recurrence versus no recurrence.

**Results:**

Thirty‐four of the 77 (44.2%) recurrences were classified as late. Among the 77 patients who recurred, smaller tumor volume (OR 0.243, *p* = 0.035) and lower novel Dulguerov staging (OR 0.802, *p* = 0.038) were associated with late, rather than early recurrence. When patients with late recurrence were compared to those who remained disease‐free, radiographic skull base bone involvement was the strongest independent predictor (OR 2.90, 95% CI 1.14–8.64, *p* = 0.025), and this effect strengthened after adjusting for tumor volume (OR 3.80, *p* = 0.029). Pathologic dural invasion (OR 3.29, *p* = 0.013), Kadish stage (OR 2.02, *p* = 0.026), and T‐stage (OR 1.32, *p* = 0.040) were also significant.

**Conclusions:**

A 48‐month cutoff effectively stratifies ONB recurrence risk. Skull base bone involvement independently predicts recurrence when compared to nonrecurrent cases and uniquely persists as a risk factor for late recurrence. These findings establish skull base bone involvement as a novel biomarker for risk‐stratified surveillance protocols.

## 1 Introduction

Olfactory neuroblastoma (ONB), also known as esthesioneuroblastoma, is a rare malignant neuroectodermal tumor arising from the olfactory epithelium of the superior nasal cavity, accounting for 3%–6% of sinonasal cancers and with an annual incidence of 0.4–1.0 per million people [1, 2, 3, 4, 5]. Despite its rarity, ONB has garnered notable clinical and research attention due to its unique biological behavior, characterized by a well‐documented tendency toward late recurrence, distinguishing it from most head and neck malignancies [6, 7, 8, 9].

Unlike squamous cell carcinomas of the head and neck, where most recurrences occur within the first 2–3 years after treatment, ONB shows recurrence patterns that extend well beyond typical surveillance periods [10, 11, 12]. Studies have documented recurrences at 10, 15, and even 20 years after initial treatment, with approximately one‐third occurring after the standard 5‐year follow‐up [13, 14, 15, 16]. Delayed cervical nodal and distant metastases further complicate long‐term management [17, 18]. This unpredictable timing challenges both clinical surveillance planning and patient counseling [11].

Current surveillance guidelines for ONB differ significantly among institutions, with recommendations spanning from 5 years to lifelong follow‐up [11, 19, 20]. Recent expert consensus statements from the American Rhinologic Society have highlighted the importance of extended surveillance, although specific risk‐stratification criteria remain poorly defined [12]. This variability reflects a fundamental gap in our knowledge: although many studies have identified predictors of overall recurrence risk, the factors that distinguish patients at risk for early versus late recurrence are still largely unexplored.

Multiple staging systems have been developed for ONB, including the Kadish, modified Kadish, Dulguerov, and American Joint Committee on Cancer (AJCC) systems [21, 22, 23, 24, 25]. The Hyams histologic grading system provides additional prognostic stratification based on architectural and cytologic features [26, 27, 28, 29]. Recently, a proposed novel staging modification integrating Hyams grade with anatomic staging demonstrated improved prognostic discrimination for overall and disease‐free survival [30, 31, 32]. Pathologic dural invasion has also been shown to be a predictive factor for recurrence and regional metastases but has not been investigated for predicting the timing of recurrence [33].

Identifying predictors of early versus late recurrence would better inform risk‐stratified surveillance protocols, allowing de‐escalation for low‐risk patients while ensuring vigilance for those at highest risk of late recurrence. The purpose of this multi‐institutional study was to identify clinical, anatomic, and treatment factors that distinguish early from late recurrence in ONB, with particular attention to factors that predict late recurrence versus sustained disease‐free survival.

## 2 Materials and Methods

### 2.1 Study Design and Population

This multi‐institutional retrospective cohort study included consecutive patients with histologically confirmed ONB treated at nine academic medical centers in the modern treatment era between 2005 and 2021. Institutional review board approval was obtained at each participating site, with informed consent waived due to the retrospective nature of the study. Patients were included if they had primary ONB with adequate clinical documentation for staging and minimum follow‐up of 12 months for nonrecurrent cases. Patients with inadequate follow‐up, missing recurrence data, or synchronous malignancies were excluded.

### 2.2 Data Collection

Clinical, radiographic, pathologic, and treatment data were abstracted from electronic medical records using a standardized data collection instrument. Variables included patient demographics (age at diagnosis and sex), tumor characteristics (volume, Hyams histologic grade, and anatomic extent), staging (AJCC eighth edition, Kadish, modified Kadish, and Dulguerov T‐stage), and treatment factors (surgical approach, margin status, radiation therapy, chemotherapy, neck dissection, and neck radiation). Anatomic extent was characterized by radiographic assessment of the involvement of specific structures on preoperative CT and MRI, including sinus involvement (frontal, sphenoid, maxillary, and ethmoid), skull base bone, lamina papyracea, periorbita, orbital apex, cavernous sinus, dura, and brain parenchyma. Skull base bone involvement was defined as radiographic evidence of bony erosion or destruction of the anterior skull base (cribriform plate, fovea ethmoidalis, and/or planum sphenoidale) on pretreatment CT; all images were reviewed at each study site. A second reviewer was asked to review if an aspect of the imaging was not clear to come to consensus. Tumor volume was calculated from preoperative imaging using the ellipsoid formula (length × width × height × 0.52) and log‐transformed (base 10) for analysis. The novel Dulguerov staging modification, which integrates Hyams histologic grade with the Dulguerov T‐stage to create a composite prognostic score, was calculated according to previously described methodology [30].

### 2.3 Outcome Definitions

Recurrence was defined as radiographic or histologic evidence of disease after completion of initial treatment with a documented disease‐free interval. Time to recurrence was calculated from the date of definitive surgical treatment to the date of first documented recurrence. Based on the mean recurrence time observed in our cohort (47.7 months), a 48‐month cutoff was used to classify recurrences as early (< 48 months) or late (≥ 48 months). A minimum disease‐free interval of 3 months was required to distinguish true recurrence from persistent disease; patients with documented recurrence at < 3 months from definitive treatment were classified as persistent disease and excluded (*n* = 3).

### 2.4 Statistical Analysis

Descriptive statistics were calculated for all variables. Continuous variables were reported as mean ± standard deviation or median with interquartile range as appropriate. Categorical variables were reported as frequencies and percentages. Group comparisons used independent samples *t*‐tests for continuous variables and chi‐square or Fisher's exact tests for categorical variables.

Thirty‐five clinical, anatomic, and treatment variables were evaluated using Firth penalized logistic regression to account for small sample sizes and rare events. Three distinct comparisons were performed: (1) late versus early recurrence among recurrent patients, identifying predictors of recurrence timing; (2) late recurrence versus no recurrence, identifying predictors of late recurrence occurrence; and (3) early recurrence versus no recurrence, identifying predictors of early recurrence occurrence. Variables with *p *< 0.10 in univariable analysis were included for multivariable models. Final models were selected based on clinical relevance and sample size adequacy (minimum 10 events per predictor). Collinearity was assessed using variance inflation factors (VIF< 3). To address potential lead‐time bias in the nonrecurrent comparison group, two sensitivity analyses were performed: (A) excluding nonrecurrent patients diagnosed after 2018, and (B) restricting the nonrecurrent group to patients with ≥ 48 months of follow‐up. All analyses were performed using R version 4.2. Two‐sided *p* < 0.05 were considered statistically significant. The study was conducted and reported in accordance with STROBE guidelines.

## 3 Results

### 3.1 Patient Characteristics and Recurrence Patterns

Out of 258 patients assessed for eligibility across nine academic medical centers, 39 were excluded: 6 for missing or unknown recurrence data, 30 nonrecurrent patients with <12 months of follow‐up, and 3 patients with recurrence at < 3 months classified as persistent disease. The final cohort comprised 219 patients (Table 1). The mean age at diagnosis was 51.8 ± 15.3 years, and 120 (54.8%) among them were male. The majority of patients presented with advanced T‐stage disease (T3–T4, 68.0%) and Kadish stage C (62.6%). Radiographic skull base bone involvement was present in 128 of 191 patients (67.0%) with evaluable pretreatment CT imaging.

**TABLE 1:** Patient demographics, tumor characteristics, and treatment by recurrence status.

Variable	All patients (*n* = 219)	Recurrence (*n* = 77)	No recurrence (*n* = 142)
Demographics			
Female	99 (45.2%)	31 (40.3%)	68 (47.9%)
Male	120 (54.8%)	46 (59.7%)	74 (52.1%)
Age, mean ± SD	51.8 ± 15.3	51.8 ± 13.6	51.7 ± 16.1
Staging			
T1	33 (15.1%)	4 (5.2%)	29 (20.7%)
T2	25 (11.4%)	7 (9.1%)	18 (12.9%)
T3	26 (11.9%)	7 (9.1%)	19 (13.6%)
T4a	34 (15.5%)	15 (19.5%)	19 (13.6%)
T4b	89 (40.6%)	35 (45.5%)	54 (38.6%)
Early T‐stage (T1–T2)	58 (26.5%)	11 (14.3%)	47 (33.6%)
Advanced T‐stage (T3–T4)	149 (68.0%)	57 (74.0%)	92 (65.7%)
Kadish A	25 (11.4%)	4 (5.2%)	21 (15.0%)
Kadish B	49 (22.4%)	11 (14.3%)	38 (27.1%)
Kadish C	137 (62.6%)	56 (72.7%)	81 (57.9%)
AJCC Stage I	32 (14.6%)	4 (5.2%)	28 (20.1%)
AJCC Stage II	24 (11.0%)	6 (7.8%)	18 (12.9%)
AJCC Stage III	39 (17.8%)	12 (15.6%)	27 (19.4%)
AJCC Stage IVa	33 (15.1%)	16 (20.8%)	17 (12.2%)
AJCC Stage IVb	78 (35.6%)	29 (37.7%)	49 (35.3%)
Hyams grade			
Grade 1	13 (8.2%)	4 (7.1%)	9 (8.8%)
Grade 2	84 (53.2%)	26 (46.4%)	58 (56.9%)
Grade 3	48 (30.4%)	17 (30.4%)	31 (30.4%)
Grade 4	13 (8.2%)	9 (16.1%)	4 (3.9%)
Low (1–2)	97 (61.4%)	30 (53.6%)	67 (65.7%)
High (3–4)	61 (38.6%)	26 (46.4%)	35 (34.3%)
(evaluable)	158/219	56/77	102/142
Anatomic involvement			
Skull base bone	128/191 (67.0%)	53/63 (84.1%)	75/128 (58.6%)
Dura (radiographic)	89/188 (47.3%)	39/61 (63.9%)	50/127 (39.4%)
Brain	41/186 (22.0%)	19/61 (31.1%)	22/125 (17.6%)
Periorbita	36/187 (19.3%)	19/62 (30.6%)	17/125 (13.6%)
Lamina papyracea	58/183 (31.7%)	26/59 (44.1%)	32/124 (25.8%)
Orbital apex	10/186 (5.4%)	4/60 (6.7%)	6/126 (4.8%)
Treatment			
Endoscopic approach	136 (62.1%)	39 (50.6%)	97 (68.8%)
Open approach	26 (11.9%)	13 (16.9%)	13 (9.2%)
Combined approach	48 (21.9%)	20 (26.0%)	28 (19.9%)
Any radiation	126/160 (78.8%)	42/54 (77.8%)	84/106 (79.2%)
Chemotherapy	59/217 (27.2%)	29/76 (38.2%)	30/141 (21.3%)
Positive margins	49/187 (26.2%)	23/59 (39.0%)	26/128 (20.3%)
Recurrence (48‐month cutoff)			
Any recurrence	77 (35.2%)	77 (100%)	—
Early recurrence (< 48 month)	43 (55.8%^a^)	43 (55.8%)	—
Late recurrence (≥ 48 month)	34 (44.2%^a^)	34 (44.2%)	—

Abbreviations: CRT = chemoradiation, XRT = radiation therapy.

^a^ Percentage of recurrent patients.

Median follow‐up for the entire cohort was 61.3 months (IQR 36.2–104.6). Seventy‐seven patients (35.2%) developed recurrence during follow‐up, including 43 early recurrences (55.8%) and 34 late recurrences (44.2%). Among recurrent patients, the mean time to first recurrence was 49.6 ± 41.0 months (median 42.0 months, range 3.0–178.0 months). Among nonrecurrent patients (*n* = 142), the median follow‐up was 56.0 months (IQR 33.7–89.6), and 80 (56.3%) had ≥ 48 months of follow‐up. The distribution of recurrence timing is shown in Figure 1.

**FIGURE 1:**
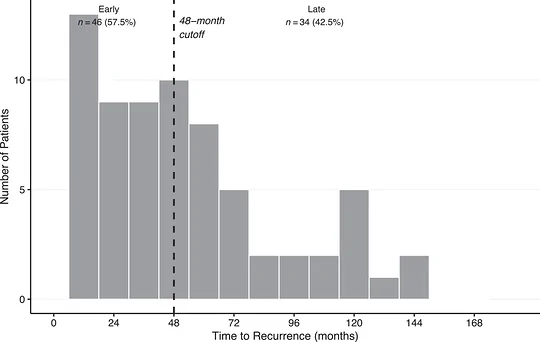
Distribution of time to recurrence in olfactory neuroblastoma. Histogram of recurrence timing among the 80 patients who experienced recurrence, with the 48‐month cutoff demarcated by a vertical dashed line. Forty‐six patients (57.5%) experienced early recurrence (< 48 months) and 34 (42.5%) experienced late recurrence (≥ 48 months).

Among 77 recurrent patients, local recurrence was most common (53.2%), followed by neck (44.2%), intracranial (10.4%), and distant (9.1%). The distribution of recurrence sites did not differ between early and late groups at any site (all *p *> 0.29), with local recurrence predominating in both (51.2% vs. 55.9%, *p* = 0.819). Patients with skull base bone involvement had larger primary tumors (mean log volume: 4.52 vs. 4.04, *p* < 0.001) and higher novel Dulguerov stage (mean: 5.16 vs. 2.39, *p *< 0.001) than those without skull base bone involvement.

### 3.2 Predictors of Recurrence Timing (Late vs. Early Recurrence)

Among the 77 patients with tumor recurrence, five variables significantly predicted recurrence timing in univariable analysis (Table 2). Smaller tumor volume (OR 0.243 per log‐unit increase, 95% CI 0.053–0.907, *p* = 0.035) and lower novel Dulguerov staging (OR 0.802 per unit increase, 95% CI 0.641–0.988, *p* = 0.038) were associated with late recurrence. Ethmoid sinus involvement (OR 0.329, 95% CI 0.117–0.854, *p* = 0.022), maxillary sinus involvement (OR 0.410, 95% CI 0.138–0.996, *p* = 0.049), and neck radiation (OR 0.438, 95% CI 0.188–0.874, *p* = 0.018) were all associated with early recurrence.

**TABLE 2:** Univariable predictors of late versus early recurrence timing (*n* = 80 recurrent patients).

Variable	Type	*n*	OR (95% CI)	*p*‐value
Neck radiation	Numeric	55	0.438 (0.188–0.874)	0.018
Ethmoid sinus	Numeric	66	0.329 (0.117–0.854)	0.022
Tumor volume (log)	Numeric	45	0.243 (0.053–0.907)	0.035
Novel Dulguerov	Numeric	53	0.802 (0.641–0.988)	0.038
Maxillary sinus	Numeric	63	0.410 (0.138–0.996)	0.049
Skull base bone	Binary	63	0.714 (0.189–2.688)	0.612

*Note*: OR < 1 indicates association with late recurrence. Firth penalized logistic regression.

Notably, skull base bone involvement (OR 0.714, *p* = 0.612), traditional staging systems, Hyams grade, and surgical margin status did not distinguish early from late recurrence (all *p *> 0.05). In multivariable analysis, neither tumor volume (*p* = 0.176) nor novel Dulguerov staging (s = 0.242) remained independently significant (VIF = 2.8), suggesting collinearity.

### 3.3 Predictors of Late Recurrence (Late Recurrence vs. No Recurrence)

In the comparison of 34 patients with late recurrence to 142 patients without recurrence (*n* = 176), four variables significantly predicted late recurrence in univariable analysis (Table 3): pathologic dural invasion (OR 3.287, 95% CI 1.278–9.395, *p* = 0.013), skull base bone involvement (OR 2.899, 95% CI 1.138–8.642, *p* = 0.025), Kadish stage (OR 2.016, 95% CI 1.082–4.303, *p* = 0.026), and T‐stage (OR 1.319, 95% CI 1.012–1.771, *p* = 0.040). Radiographic dural involvement also trended toward significance (OR 2.149, 95% CI 0.965–4.911, *p* = 0.061). Tumor volume (*p* = 0.618), novel Dulguerov staging (*p* = 0.540), and other anatomic, treatment, and histologic variables were not significant (all *p *> 0.05).

**TABLE 3:** Predictors of late recurrence versus no recurrence (*n* = 206).

Variable	Type	*n*	OR (95% CI)	*p*‐value
Pathologic dural invasion^a^	Binary	130	3.287 (1.278–9.395)	0.013
Skull base bone^a^	Binary	155	2.899 (1.138–8.642)	0.025
Kadish	Numeric	171	2.016 (1.082–4.303)	0.026
T‐stage	Numeric	170	1.319 (1.012–1.771)	0.040
Dura (radiographic)	Binary	156	2.149 (0.965–4.911)	0.061

^a^ Significant predictors. Multivariable: Skull base bone adjusted for tumor volume: OR 3.798, *p* = 0.029 (*n* = 115); adjusted for novel Dulguerov: OR 3.491, *p* = 0.052 (*n* = 116). Collinearity: SBB + pathologic dural invasion in the same model: SBB OR 1.715 (*p* = 0.451), PathDI OR 2.043 (*p* = 0.212), *n* = 120. Fisher's exact OR 23.82, *p *< 0.001, concordance 75.8%.

In multivariable analysis, the effect of skull base bone involvement strengthened when adjusted for tumor volume (OR 3.798, *p* = 0.029; *n* = 115) and was borderline after adjusting for novel Dulguerov staging (OR 3.491, *p* = 0.052; *n* = 116). Skull base bone involvement was strongly associated with pathologic dural invasion (Fisher's exact OR 23.82, *p *< 0.001; 75.8% concordance). When both variables were included in the same multivariable model, neither retained independent significance (skull base bone OR 1.715, *p* = 0.451; pathologic dural invasion OR 2.043, *p* = 0.212; *n* = 120), confirming collinearity between these measures of skull base invasion. The pattern of skull base bone involvement across all comparisons is illustrated in Figure 2 and this pattern is further summarized in Table 4.

**FIGURE 2:**
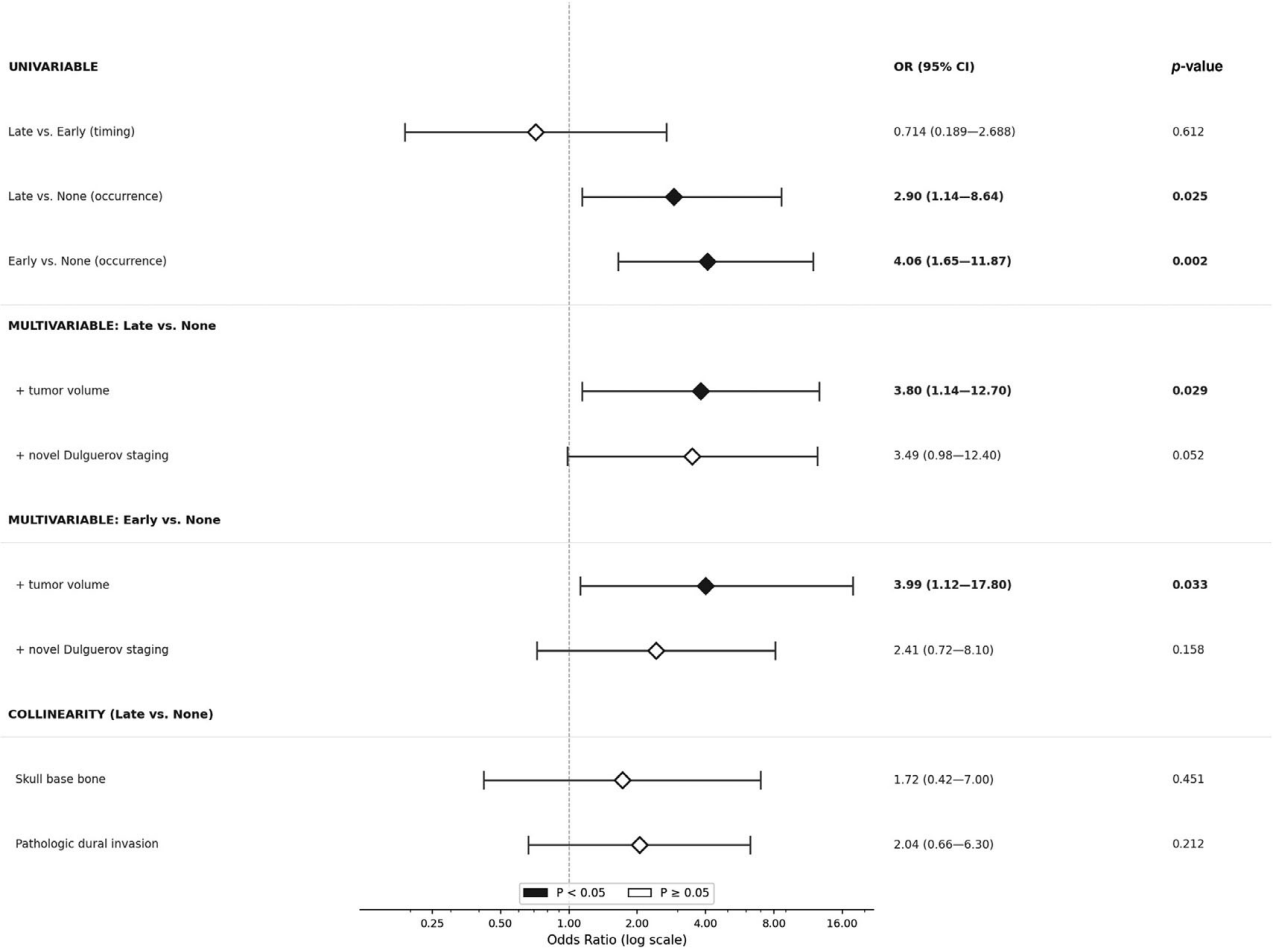
Forest plot of skull base bone involvement as a predictor of recurrence across univariable, multivariable, and collinearity analyses. In univariable analysis, skull base bone involvement did not distinguish early from late recurrence among patients who recurred (odds ratio [OR] 0.71, *p* = 0.612) but significantly predicted both late (OR 2.90, *p* = 0.025) and early (OR 4.06, *p* = 0.002) recurrence versus no recurrence. In multivariable models for late recurrence versus no recurrence, the effect of skull base bone involvement strengthened after adjusting for tumor volume (OR 3.80, *p* = 0.029) and was borderline after adjusting for novel Dulguerov staging (OR 3.49, *p* = 0.052). In the parallel analysis for early recurrence, skull base bone involvement remained significant after adjusting for tumor volume (OR 3.99, *p* = 0.033) but was attenuated after adjusting for novel Dulguerov staging (OR 2.41, *p* = 0.158). When skull base bone involvement and pathologic dural invasion were entered in the same model, neither remained significant (*p* = 0.451 and *p* = 0.212, respectively; *n* = 120), consistent with collinearity between the two variables. Filled diamonds denote statistical significance (*p *< 0.05) and open diamonds denote non‐significance. Error bars represent 95% confidence intervals. All models were fit with Firth penalized logistic regression.

**TABLE 4:** Skull base bone involvement: pattern across all three comparisons.

Comparison	OR (95% CI)	*p*‐value	Significant?
Late vs. early (timing)	0.714 (0.189–2.688)	0.612	No
Late vs. none (occurrence)	2.899 (1.138–8.642)	0.025	Yes
Early vs. none (occurrence)	4.058 (1.645–11.874)	0.002	Yes

Patients with skull base bone involvement received significantly more radiation therapy (89.9% vs. 64.6%, *p *< 0.001) and chemotherapy (35.4% vs. 12.7%, *p* = 0.001). Despite receiving more intensive treatment, skull base bone involvement remained independently associated with late recurrence. Positive surgical margins predicted early recurrence (OR 3.426, *p* = 0.003) but not late recurrence (OR 1.686, *p* = 0.272). Patients with skull base bone involvement had significantly higher rates of positive margins (35.9% vs. 10.9%, *p *< 0.001).

### 3.4 Predictors of Early Recurrence (Early Recurrence vs. No Recurrence)

In the comparison of 43 patients with early recurrence to 142 patients without recurrence (*n* = 185), 18 variables significantly predicted early recurrence (Table 5). The strongest predictors included novel Dulguerov staging (OR 1.327, 95% CI 1.127–1.583, *p* = 0.001), skull base bone involvement (OR 4.058, 95% CI 1.645–11.874, *p* = 0.002), tumor volume (OR 3.833, 95% CI 1.528–10.874, *p* = 0.003), and positive surgical margins (OR 3.426, 95% CI 1.525–7.718, *p* = 0.003). Additional significant predictors encompassed multiple staging systems, anatomic involvement variables, Hyams grade, and surgical approach.

**TABLE 5:** Significant predictors of early recurrence versus no recurrence (*n* = 218).

Variable	Type	*n*	OR (95% CI)	*p*‐value
Novel Dulguerov	Numeric	131	1.327 (1.127–1.583)	0.001
Skull base bone	Binary	164	4.058 (1.645–11.874)	0.002
Tumor volume (log)	Numeric	121	3.833 (1.528–10.874)	0.003
Dura (radiographic)	Binary	159	3.289 (1.488–7.680)	0.003
Margins (positive)	Binary	160	3.426 (1.525–7.718)	0.003
Dulguerov T‐stage	Numeric	180	1.610 (1.203–2.215)	0.001
Periorbita	Binary	157	4.292 (1.811–10.189)	0.001
T‐stage	Numeric	182	1.453 (1.138–1.911)	0.002
Ethmoid sinus	Numeric	168	2.710 (1.421–5.413)	0.002
Hyams (dichotomized)	Binary	133	2.166 (1.271–3.817)	0.004
Lamina papyracea	Binary	157	3.019 (1.381–6.662)	0.006
AJCC overall stage	Numeric	179	1.391 (1.084–1.826)	0.009
Sphenoid sinus	Numeric	160	1.744 (1.122–2.720)	0.014
Kadish	Numeric	180	1.951 (1.114–3.764)	0.018
Brain	Binary	157	2.805 (1.197–6.471)	0.018
Modified Kadish	Numeric	181	1.583 (1.042–2.485)	0.031
Pathologic dural invasion	Binary	139	2.308 (1.036–5.354)	0.041
Surgery (approach)	Numeric	182	1.463 (1.003–2.130)	0.048

*Note*: All 18 significant predictors are shown (*p *< 0.05). Firth penalized logistic regression. Multivariable: Skull base bone adjusted for tumor volume: OR 3.994, *p* = 0.033 (*n* = 119); adjusted for novel Dulguerov: OR 2.414, *p* = 0.158 (*n* = 124).

In multivariable analysis for early recurrence, skull base bone involvement remained significant after adjusting for tumor volume (OR 3.994, *p* = 0.033; *n* = 119) but was attenuated after adjusting for novel Dulguerov staging (OR 2.414, *p* = 0.158; *n* = 124). Novel Dulguerov staging (*p* = 0.001) and tumor volume (*p* = 0.003) both retained independent significance in their respective adjusted models.

### 3.5 Sensitivity Analyses

Two sensitivity analyses were performed to evaluate the robustness of findings to potential follow‐up bias in the nonrecurrent comparison group. In sensitivity analysis A, excluding nonrecurrent patients diagnosed after 2018 who could not have achieved 48 months of follow‐up (*n* = 204), the skull base bone involvement effect remained significant across all models: univariable OR 3.092 (95% CI 1.205–9.268, *p* = 0.018), volume‐adjusted OR 3.784 (*p* = 0.028), and novel Dulguerov‐adjusted OR 3.700 (*p* = 0.041). In Sensitivity Analysis B, restricting the nonrecurrent group to patients with ≥ 48 months of follow‐up (*n* = 114), the effect strengthened further: univariable OR 3.470 (95% CI 1.297–https://doi.org/10.727, *p* = 0.012), volume‐adjusted OR 4.400 (*p* = 0.021), and novel Dulguerov‐adjusted OR 4.217 (*p* = 0.037). The consistency of these findings across analytic approaches is illustrated in Figure 3.

**FIGURE 3:**
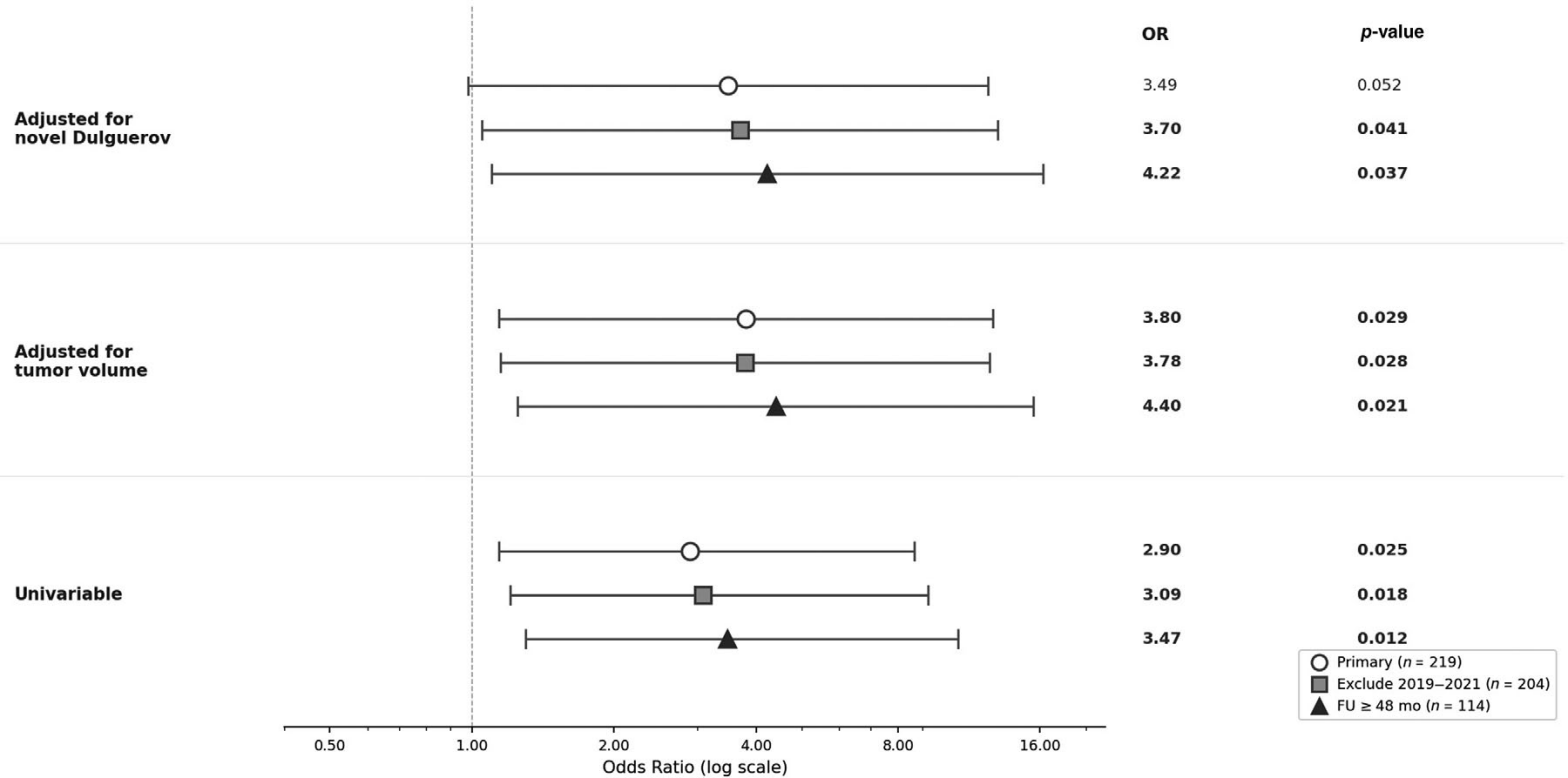
Sensitivity analyses for skull base bone involvement as a predictor of late recurrence versus no recurrence. The effect of skull base bone involvement is shown across three analytic approaches: the primary analysis (*n* = 219), sensitivity analysis A excluding nonrecurrent patients diagnosed after 2018 (*n* = 204), and sensitivity analysis B restricting the nonrecurrent group to patients with ≥ 48 months of follow‐up (*n* = 114). Results are shown for univariable, volume‐adjusted, and novel Dulguerov‐adjusted models. The effect of skull base bone involvement strengthened progressively with more rigorous follow‐up restriction, with odds ratios ranging from 2.90 to 4.40. All models reached statistical significance across both sensitivity analyses, including the novel Dulguerov‐adjusted model (*p* = 0.041 and *p* = 0.037), which had been borderline in the primary analysis (*p* = 0.052). Error bars represent 95% confidence intervals. All models were fit with Firth penalized logistic regression.

## 4 Discussion

This multi‐institutional study of 219 ONB patients treated at nine academic centers provides novel insights into the biology and clinical management of early versus late recurrence. The principal finding is that skull base bone involvement independently predicts late recurrence after adjusting for tumor burden. While multiple tumor biology markers are associated with early recurrence, skull base bone involvement uniquely retains prognostic weight for late recurrence. This distinction positions skull base bone involvement as a clinically actionable biomarker for risk‐stratified surveillance protocols.

### 4.1 Dissociation Between Predictors of Timing and Occurrence

The most notable finding from our analysis is the disconnect between predictors of recurrence timing and those of late recurrence. Early recurrence was predicted by 18 variables spanning tumor burden, anatomic extent, staging, histologic grade, and treatment factors, reflecting the multifactorial biology of aggressive early treatment failure. In contrast, late recurrence was predicted by a narrower set of variables including skull base bone involvement, pathologic dural invasion, T‐stage, and Kadish stage, all reflecting the degree of skull base invasion rather than overall tumor aggressiveness. Tumor volume and novel Dulguerov staging strongly predicted whether patients would recur (early vs. late), but they did not distinguish patients likely to have late recurrence from those who remained disease‐free.

This pattern suggests distinct biological mechanisms: early recurrence appears driven by aggressive tumor biology (large size, high grade, and extensive invasion), whereas late recurrence may be attributable to anatomic sanctuary sites that allow long‐term disease persistence. Multivariable analysis revealed a critical distinction. For late recurrence versus no recurrence, skull base bone involvement strengthened after adjusting for tumor volume (OR 3.80, *p* = 0.029), whereas tumor volume and novel Dulguerov staging did not independently predict late recurrence. This pattern was not observed for early recurrence, where both skull base bone involvement and tumor volume retained independent significance. The differential behavior emerged most clearly when adjusting for the novel Dulguerov composite stage: skull base bone involvement was attenuated for both late (*p* = 0.052) and early (*p* = 0.158) recurrence, while the composite stage retained significance only for early recurrence (*p* = 0.001 vs. *p* = 0.540), suggesting it captures tumor biology driving early recurrence but not the sanctuary mechanism underlying late recurrence.

### 4.2 Skull Base Bone as a Sanctuary Site

We hypothesize that skull base bone may act as a “sanctuary site” harboring microscopic residual disease that evades both surgical resection and adjuvant therapy. The osseous microenvironment could offer protection from treatment while permitting slow tumor growth over extended periods, a phenomenon consistent with models of tumor dormancy in bone [34, 35, 36, 37]. Unlike soft‐tissue recurrences, which tend to appear earlier, bone‐resident disease might remain dormant for years before becoming clinically evident. Despite preoperative identification and targeted treatment, microscopic disease may persist within the trabecular bony matrix or along bone‐dura interfaces, where drilling and radiation doses are constrained by adjacent neurovascular structures. Whether more aggressive bony resection could reduce late recurrence risk warrants prospective investigation.

Several observations support this idea. First, skull base bone involvement predicted late recurrence regardless of tumor size and stage, indicating a qualitative rather than quantitative effect. Second, while skull base bone involvement predicted both early and late recurrence in univariable analysis, its prognostic behavior diverged in multivariable models. After adjusting for tumor volume, skull base bone involvement remained significant in both comparisons; however, the integrated novel Dulguerov staging attenuated the effect of skull base bone involvement for early recurrence but not for late recurrence when volume alone was adjusted. This indicates that for early recurrence, skull base bone involvement largely functions as a marker of overall tumor burden, whereas for late recurrence, it carries independent prognostic information beyond what is captured by tumor biology.

Third, skull base bone involvement was strongly collinear with pathologic dural invasion (Fisher's exact OR 23.82, *p *< 0.001; 75.8% concordance). Notably, pathologic dural invasion was also independently significant for late recurrence in univariable analysis (OR 3.287, *p* = 0.013). When both variables were included in the same multivariable model, neither retained significance (skull base bone OR 1.715, *p* = 0.451; dural invasion OR 2.043, *p* = 0.212; *n* = 120), confirming that they measure overlapping aspects of skull base invasion biology. Skull base bone involvement, assessed on preoperative imaging, thus serves as a practical radiographic surrogate for pathologic skull base invasion, enabling prospective surveillance planning. This may be complicated by a bias toward dural resection in cases of bony invasion; however, bony invasion is more reliably assessed radiographically. The distribution of recurrence sites further supports this hypothesis: local recurrence predominated in both early and late groups with no significant difference at any site, arguing against delayed distant metastasis and for local disease persistence at the skull base.

### 4.3 Implications for Surveillance

Our finding that over 44% of recurrences happen beyond 4 years highlights the inadequacy of traditional 5‐year surveillance protocols for head and neck malignancies. This rate exceeds previous estimates of 25%–35%, likely reflecting longer follow‐up and systematic multi‐institutional case ascertainment [13, 14, 15, 16]. These data strongly support extended surveillance beyond 5 years for all ONB patients, consistent with prior institutional series and recent expert consensus [12, 38]. Based on our findings, we recommend a minimum of 10 years of clinical follow‐up for all ONB patients, with selective imaging after 5 years. Patients with skull base bone involvement warrant indefinite surveillance given their nearly threefold elevated risk. Recent ARS consensus has highlighted somatostatin receptor‐based imaging (e.g., 68Ga‐DOTATATE PET/CT) for sinonasal malignancy surveillance, which may be particularly relevant for detecting recurrence at the bony skull base [12]. Imaging modalities capable of evaluating bone, including CT and PET/CT, should be strongly considered in the extended surveillance paradigm. Late recurrence site distribution further supports this hypothesis: local recurrence predominated and typically occurred in isolation, while late neck recurrences largely occurred independently of local disease, likely reflecting a separate mechanism of delayed regional metastasis. Skull base bone involvement may predispose to both.

### 4.4 Limitations

Several limitations should be considered. First, the retrospective multi‐institutional design may introduce selection and information biases, and missing data reduced statistical power for some variables. Second, the small number of late recurrence cases (*n* = 34) restricts the precision of effect estimates and limits the complexity of multivariable analyses. Finally, external validation in an independent cohort is necessary before risk‐stratified surveillance can be broadly applied in clinical settings.

## 5 Conclusions

Over 44% of ONB recurrences happen after 4 years, challenging traditional surveillance strategies. Our study is suggestive of different mechanisms for early and late recurrence: early recurrence is associated with multiple tumor burden markers, including tumor volume, histologic grade, and positive margins, whereas late recurrence is associated with markers of skull base invasion, most notably skull base bone involvement, which retains independent predictive value after adjusting for tumor burden. We recommend a minimum of 10 years of clinical follow‐up for all ONB patients, with indefinite surveillance for those with skull base bone involvement, who face nearly a threefold higher risk of late recurrence. Conversely, patients without skull base bone involvement might consider less intensive surveillance after 5 years without disease. These results position skull base bone involvement as a new biomarker for risk‐based surveillance strategies in olfactory neuroblastoma.

## Conflicts of Interest

The authors declare no conflicts of interest.
